# Reflux, Intraocular Pressure Variation and Pain Following Intravitreal Ranibizumab Injections Using 30-Gauge or 32-Gauge Needles for Patients With Retinal Pathologies: A Randomized Clinical Trial

**DOI:** 10.7759/cureus.14320

**Published:** 2021-04-06

**Authors:** Saeed T Alshahrani, Uriel Rubin, Vasudha Gupta, Tom Gonder, Sanjay Sharma

**Affiliations:** 1 Ophthalmology Department, King Fahad Medical City, Riyadh, SAU; 2 Ophthalmology, Queens University, Kingston, CAN

**Keywords:** intravitreal injection, intra ocular pressure, retinal diseases, retina, vitreous

## Abstract

Purpose: To compare reflux, intraocular pressure (IOP) variation and pain following intravitreal (IV) injections using 30-gauge and 32-gauge needles in patients with retinal pathologies in Saudi Arabia.

Methods: A double-blind randomized clinical trial was conducted in 2018. Participants were randomized to receive IV injections of Ranibizumab using 30-gauge (Gr_1_) or 32-gauge (Gr_2_) needles. The amount of reflux of injected material, IOP before (IOP_1_) and five minutes after injection (IOP_2_) were measured. The patient-perceived pain score was assessed using a visual analogue score (VAS). Outcome variables were compared.

Results: The study sample was comprised of 86 eyes (86 patients) in each group. Gender (P=0.76), laterality (P=0.55) and age (P=1.0) were not different between groups. The reflux in Gr_1_ [34.9% (95% confidence interval {CI}, 24.8; 45.0)] was significantly higher compared to Gr_2_ [22.1% (95% CI, 13.3; 30.9)] (P=0.007). The median pain score was 1 in both Gr_1_ [interquartile range (IQR) 1.0: 3.0] and Gr_2_ (IQR 0.0; 2.0) (P=0.04). Among 56 eyes without reflux in Gr_1_, the IOP_1_ and IOP_2_ were 13.6±2.7 mmHg and 16.4±5.0 mmHg, respectively. Among 67 eyes without reflux in Gr_2_, the IOP_1_ and IOP2 were 13.6±2.9 mmHg and 17.0±5.2 mmHg, respectively. The change in percentage in IOP in Gr_1_ and Gr_2_ was not significantly different (Mann Whiney P=0.3).

Conclusions: IV injection given by 30-gauge needle compared to 32-gauge needle resulted in greater patient-perceived pain and more reflux of injected material from the injection site. An increase in IOP was not associated with the gauge of the needle used for IV injection.

## Introduction

Anti-vascular endothelial growth factor (VEGF) compounds are the initial treatment for diabetic macular edema (DME) with foveal involvement. These compounds can be used as isolated treatment or combined with photocoagulation [1,2]. Intravitreal (IV) injections of anti-VEGF compounds are performed on an outpatient basis for a number of other retinal pathologies [3,4]. IV injections of anti-VEGF compounds are known to cause short-term and sustained increases in intraocular pressure (IOP) [5,6]. Therefore, IOP monitoring is recommended soon after and at regular intervals following IV injections [6]. Patient compliance is vital for treatments that require repeat injections. Hence, patient-perceived discomfort and pain during intravitreal injections should be assessed and addressed as warranted [7-9]. For optimum availability of anti-VEGF medication in the vitreous cavity proximal to the site of pathology, different routes, different sites and different sized injections (gauge) have been investigated [10-12]. However, reflux occurs soon after intravitreal injection and it contains the injected material and vitreous. Fortunately, less than 1% of injected material is lost with reflux [13]. The reflux can vary by size of entry in the sclera and other layers of the globe depending on the needle gauge.

To the best of our knowledge, studies have compared outcomes of intravitreal injections using the different gauge of needles for 27 vs 30 and 30 vs 33 gauge needles but not 30 vs 32 gauge needles in Arab patients [11,12].

We present the results of a double-masked randomized clinical trial (RCT) comparing the reflux, short-term IOP and the patient-perceived pain among separate groups of patients managed with 30-gauge vs 32-gauge needle for IV injection for different retinal pathologies.

## Materials and methods

Patients with macular edema due to diabetic retinopathy, exudative age-related macular edema or retinal vasculopathy visiting our institution for intravitreal anti-VEGF injection comprised the study population. The institution research and ethics committee approved this study and this study adhered to the tenets of the Declaration of Helsinki. Written informed consent was obtained from each participant.

The field component of the study was carried out between January 2016 and December 2017. We included patients with retinal diseases mainly related to diabetes like DME and retinal vein occlusion needing IV injection. We excluded all not consenting to participate in the study.

We assumed that the conjunctival reflux would occur in 11.3% of eyes that received IV injection with a 30-gauge needle whereas the reflux rate was in 1% of eyes treated with a 32-gauge needle [13]. To achieve a 95% confidence interval (CI) and 80% power for this RCT with a 1:1 ratio for random allocation in each group, at least 86 eyes were required. The sample size was calculated with Open epi software [14].

Patients were randomly allocated to treatment with a 30-gauge needle (Gr1) or with a 32-gauge needle (Gr2). The Microsoft XL®’s random sample function was used to allocate patients in two arms of the study. The patient was not aware of the needle gauge used for intravitreal injection. The data analyzer was also masked about Gr1 and Gr2 identity.

Patients with macular edema agreeing to undergo management by IV injection of anti-VEGF compounds were included in the study. Patients were excluded if they declined to participate, had received intravitreal injection previously or experienced a painful ocular condition in addition to macular edema.

Demographic data were collected including patient age at presentation and gender. A retina specialist diagnosed the retinal pathology causing macular edema. The potential causes included age-related macular degeneration (AMD), DME or other retinal pathologies. The best-corrected visual acuity (BCVA) was recorded with ETDRS distance vision charts. Visual impairment was further graded as; normal functional vision (20/20 to 20/50), moderate visual impairment (<20/60 to 20/200), severe visual impairment (<20/200 to 20/400) and blind (<20/400) [15].

The procedure for IV injection used in the present study has been previously published [1,16]. Briefly, the eye was cleansed, and povidone-iodine and xylocaine gel were instilled, after 10 minutes an eye speculum was inserted, and povidone-iodine was instilled again. All intravitreal injections were performed by a retina specialist. The patient was asked to sit on an ophthalmic chair with chin up in a minor surgical procedure room. A mark at a distance of 3.5 cm from the limbus was placed in the inferotemporal quadrant to inject 0.05 ml containing 0.5 mg ranibizumab in the vitreous cavity.

Reflux was evaluated when we inject and we notice under the magnification of the operating microscope that some fluids get out from the injection site after we removed the needle which is considered as +ve reflux. The IOP was measured before and five minutes after IV injection using applanation XL (Medtronic, Minneapolis, MN). The difference in IOP before and five minutes after injection was used to calculate the percentage proportion of IOP change.

A visual analogue scale (VAS) was used to assess the patient-perceived severity of pain with a score from 1 to 10 [17].

Data were collected using a pretested data collection form, audited and then transferred to an Excel spreadsheet (Microsoft Corp., Redmond, WA). Data analysis was performed using Statistical Package for Social studies (SPSS 25; IBM Corp., Armonk, NY). For qualitative variable outcomes, univariate analysis with a parametric method was performed and frequencies and percentage proportions were calculated. For quantitative variables, the mean and standard deviation were reported for variables with a normal distribution. For a non-normal distribution, we estimated the median and interquartile range (IQR). To compare the pain score and IOP in two groups, we used a nonparametric method and estimated a two-sided P-value with the Mann-Whitney U test. The ordinal regression model was used to study the interaction of factors on the correlation of pain score to the gauge of the needle used for IV injection. A P-value less than 0.05 was considered statistically significant.

## Results

We included 86 eyes of 86 patients in each group. The demographic and preoperative vision profile between groups was not significantly different (Table 1).

**Table 1:** Comparison of demographic and ocular profile before intravitreal injection using 30- and 32-gauge needle. AMD: age-related macular degeneration, DME: diabetic macular edema, SDV: standard deviation.

		Gr1 (30-gauge needle; n = 86)		Gr2 (32-gauge needle; n = 86)		Validation
		Number	Percentage	Number	Percentage	
Gender	Male	50	58.1	52	60.5	P = 0.76
	Female	36	41.9	34	39.5	
Laterality	Right eye	41	47.7	45	52.3	P = 0.55
	Left eye	45	52.3	41	47.7	
Vision impairment	20/20 to 20/60	5	8.9	9	13.4	P = 0.56
	<20/60 to 20/200	34	60.7	45	67.2	
	<20/200 to 20/400	7	12.5	2	3	
	<20/400	7	12.5	10	14.9	
	Missing	3	5.4	1	1.5	
Diagnosis	AMD	54	62.8	54	62.8	P = 1.0
	DME	32	37.2	32	37.2	
Age	Mean	77.8		77.8		P = 1.0
	SDV		10.7		10.7	

Reflux was noted in 30 [34.9% (95% CI, 24.8:45.0)] eyes of Gr1 and 19 [22.1% (95% CI 13.3: 30.9)] eyes of Gr2. The reflux seems to be similar in Gr1 and Gr2 (P = 0.07).

Although the median of the pain score was 1 in both groups, the upper limit of IQR was more in Gr1 compared to that of Gr2. By using the nonparametric method of analysis, we noted that pain score was statistically significantly lower in Gr2 compared to Gr1 (Mann Whitney U test P =0.04).

**Figure 1:**
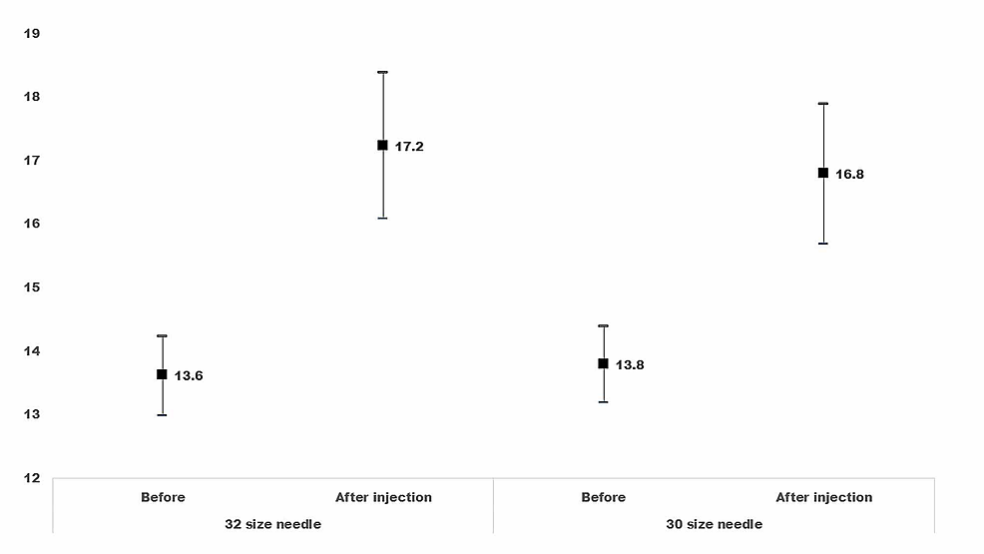
Intraocular pressure before and five minutes after intravitreal injection of anti-vascular growth factor (Ranibizumab) to treat macular edema. The X-axis shows a 30-gauge needle (Gr1) used and a 32-gauge needle (Gr2) used for intravitreal injection and IOP before vs five minutes after intravitreal injection. The Y-axis shows IOP in mmHg. In the bar graph, the central symbol denotes the mean IOP value while two ends of the bar denote the lower and upper end of the 95% confidence interval.

The IOP before and after IV injection is presented in Figure 1. The IOP increased five minutes after IV injection. However, there was no significant difference in IOP between groups. The median percentage difference in IOP before and after injection was 20% (IQR, 6: 38.5) for Gr1 and 22.5% (IQR, 5.8; 47.8) for Gr2. The percentage difference in IOP between groups was not significant (MW, P = 0.4). In Gr1, the IOP five minutes after IV injection was greater than 22 mmHg in 9 (10.5%) eyes. In Gr2 12 (14%) eyes had an IOP greater than 22 mmHg, five minutes after IV injection.

Data on the change in IOP and pain score in eyes without reflux were analyzed. There were 56 eyes in Gr1 and 67 eyes in Gr2 that did not have reflux after IV injection. In these eyes, the median pain score in Gr1 was 1.0 (IQR 0.25: 3.0) and 1.0 (IQR 0.0: 2.0) in Gr2. The pain score was significantly lower in Gr2 compared to Gr1 (MW, P = 0.04). The median IOP before and after injection changed by 20% (IQR 7 32.5) in Gr1 and 25% (IQR 6; 47) in Gr2. The changes in IOP between groups were not statistically significant (MW, P = 0.4).

Using a logistic regression model, we noted that correlation of pain score to the gauge of the needle (P = 0.05) was not influenced by gender (P = 0.3), reflux (P = 0.34), age (P = 0.64) and eye involved (P = 0.9).

## Discussion

The reflux in eyes that underwent IV injection with a 30-gauge needle and 32-gauge needle for the treatment of retinal pathologies causing macular edema was not significantly different. The pain perceived by patients was significantly higher with a 30-gauge needle compared to a 32-gauge needle. The IOP measured five minutes after intravitreal injection using a 30-gauge needle was one in ten eyes, while it was one in eight eyes that were injected by using a 32-gauge needle. Female gender, older age, presence of reflux and underlying retinal pathology causing macular edema did not significantly influence the association of perceived pain to the gauge of the needle used for IV injection.

This is perhaps the first study among Arab patients showing the outcomes of intravitreal injection using the different gauge of needles. As the use of preloaded injection cartridges for intravitreal drug delivery increases, our results support the use of smaller gauge needles and they perceive less pain as therefore more likely to be preferred by patients [18]. Additionally, the rise of IOP and reflux of injected material from smaller gauge needles were similar to 30-gauge needles. This matches with the results of Guller et al. who compared 27- vs 30-gauge needles for IV injection [11]. Van Asten et al. noted that less pain in the 33-gauge needle group than the 30-gauge needle group [12]. The use of larger gauge needles increases the opening in the sclera and uvea that can cause greater pain. The introduction of fluid in the vitreous cavity with greater velocity seems to cause more discomfort.

Reflux has been noted as less than 1% in in vitro studies [19]. However, in the in vivo studies by Lemos et al. and Lemos-Reis et al., the reflux rate was 11.3% and 21.3%, respectively [13,20]. The reflux was significantly greater when a 30-gauge needle was used compared to the 32-gauge needle [21]. In our study, it was as high as one in three eyes injected with 30-gauge needles and one in five eyes injected with 32-gauge needles. Thus, if we remove the eyes with reflux, the association of the gauge of the needle to perceived pain and IOP could change. Therefore, we had estimated outcomes in eyes without reflux also.

The patient-perceived pain score in our study was on the lower scale in both groups. Proper counseling prior to IV injection could have resulted in a lower pain score. Prior counseling and female gender have been reported to influence patient-perceived pain [12]. A study with a larger sample with prior counseling may yield a perceptible difference in pain scores between the two groups.

IOP rise following 0.5 mg in 0.05 ml Ranibizumab could be due to an increase in volume or physiological alterations within the globe. This IOP rises five minutes after was marginal and not significant. Lemos et al. have reported that subconjunctival reflux contributes to lowering IOP soon after intravitreal injection [13]. A greater IOP rise was noted after triamcinolone compared to ranibizumab injection [22]. Perhaps the property of the material injected rather than retained volume in the vitreous cavity could increase IOP after intravitreal injection. We found that fewer eyes experienced an increase in IOP in both groups compared to published studies [11,13,19].

There were some limitations to our study. The volume of reflux was not quantified. All patients were treated while seated. Hence, caution is urged with generalizing our findings to patients treated while supine.

## Conclusions

With the rising population of diabetes and the trend of treating diabetic retinopathy with IV drug delivery, the selection of client-friendly but safe method is of interest to healthcare providers. The present study demonstrated that a 32-gauge needle compared to a 30-gauge needle was beneficial both for patients and service providers. The use of small-bore needles for intravitreal injection seems to reduce patient-perceived pain and maintain a lower level of side effects such as reflux and short-term rise of IOP.
